# A randomized controlled trial to evaluate performance of pigs raised in antibiotic-free or conventional production systems following challenge with porcine reproductive and respiratory syndrome virus

**DOI:** 10.1371/journal.pone.0208430

**Published:** 2018-12-06

**Authors:** Scott Dee, Jose Ezequiel Guzman, Dan Hanson, Noel Garbes, Robert Morrison, Deborah Amodie, Lucina Galina Pantoja

**Affiliations:** 1 Pipestone Applied Research, Pipestone, Minnesota, United States of America; 2 Swine Technical Services, Zoetis, Parsippany, New Jersey, United States of America; 3 College of Veterinary Medicine, University of Minnesota, St. Paul, Minnesota, United States of America; 4 Outcomes Research, Zoetis, Parsippany, New Jersey, United States of America; University of Illinois, UNITED STATES

## Abstract

The trial objective was to compare the performance and animal health parameters of pigs raised according to one of 3 antibiotic (AB) protocols: standard AB medication consisting of mass treatment on days 4 and 21 and judicious AB therapy given therapeutically thereafter as group medication in water and feed or by individual injection (group T1, N = 702); modified AB medication identical to group T1 but with mass treatment only on day 4 and without subsequent therapeutic feed medication (group T2, N = 675); or an antibiotic-free (ABF) regimen (group T3, N = 702). All pigs were vaccinated with a modified-live porcine reproductive and respiratory syndrome virus (PRRSV) vaccine 3 days after weaning. Using a seeder pig model to mimic endemic field infection dynamics, pigs were contact-challenged with virulent PRRSV lineage 1 strain 174 four weeks after vaccination. At finishing, average daily gain (ADG) and mean feed conversion ratio (FCR) were significantly better (p ≤ 0.05) for the T1 and T2 groups compared to the T3 group. There were no significant differences in post-weaning ADG and FCR between the T1 and T2 groups. Mortality and removals significantly favored (p ≤0.05) the T1 and T2 groups (20.94% and 24.89%, respectively) versus the T3 group (57.98%). Net revenue per pig was $105.43, $98.79, and $33.81 for the T1, T2 and T3 groups, respectively. Under the conditions of this study, these results indicate that in a PRRSV-endemic setting involving bacterial co-infections, an ABF production strategy may leave pigs at considerable risk of exposure to severe clinical disease and that judicious use of antibiotics can significantly improve animal health.

## Introduction

There is growing advocacy for antibiotic-free (ABF) livestock production to minimize the emergence of antibiotic-resistant food-borne pathogens and subsequent human exposure to these treatment-refractory organisms [1–4] This trend has been driven by the escalating presence of antibiotic resistance, including multi-drug resistance, among a variety of important bacterial pathogens that infect both animals and humans [5,6]. In food-animal settings, resistant pathogens include methicillin-resistant *Staphylococcus aureus* (MRSA), multi-drug resistant non-typhoidal *Salmonella*, ciprofloxacin-resistant *Campylobacter* spp, multi-drug resistant *E*. *coli*, and vancomycin-resistant enterococci [4,7–11].

It is well documented that repeated use of antibiotics (ABs) can select for drug resistance in the target pathogens [4–6]. The improper use of antibiotics (incorrect dosage, extended administration, etc.) is acknowledged to be a leading cause of antimicrobial resistance; however, the relative contributions of ABs use in humans and food animals to the development of antimicrobial resistance is poorly understood [4–6,12]. Nevertheless, Sweden banned the feeding of sub-therapeutic AB feed additives to livestock in 1985 and other European Union countries followed suit by 2006, despite allowing the use of therapeutic ABs on a short-term prescription basis, either for individual-animal or population medication [6,13]. In contrast, sub-therapeutic use of some AB growth promotants (generally considered to be doses < 200 g/ton of feed [3]) is permitted in the U.S, provided they are not classified as medically important to humans. However, because the majority of ABs in the U.S. and other non-EU countries are used in food animals [5], sub-therapeutic AB feed additives used to promote growth in poultry and livestock have understandably become an inviting target for reduction or elimination [4,6]. In some cases, complete cessation of AB use in livestock for prophylactic, therapeutic, or growth promotion purposes has been proposed, either to meet consumer demand, or as a strategy to suppress emergence of antimicrobial resistance among zoonotic pathogens [1,2,14,15]. In the U.S., the regulatory community and the pork industry have been responsive to proposals for reducing AB use in livestock by no longer allowing ABs to be used for growth promotion in animal feed and by requiring a Veterinary Feed Directive (VFD) authorizing the use of all medically important ABs [16,17]. Beginning on January 1, 2017, U.S. swine producers were required to obtain a VFD from a licensed veterinarian in order to use medically important ABs in feed, and to obtain a veterinary prescription when using medically important ABs in water [17].

The European experience following the ban on sub-therapeutic ABs in feed has been generally positive, but variable in intensive livestock settings. When use of AB growth promotants has been discontinued, the result has often been a marked increase in the use of therapeutic ABs, followed by a decline in use as modifications in management practices reduce the pressure of infectious disease [4,6]. In addition, the prevalence of AB-resistant bacterial pathogens often decrease when AB growth promotants are discontinued [4], stimulating the interest in the development of antibiotic-free livestock production systems. While good conceptually, data from ABF production systems indicate that AB-resistant pathogens can be found in ABF swine herds, indicating that exposure to ABs is not a necessary condition for the emergence of resistance [10,11,18,19]. In addition, definitive data on the effect of this strategy on production performance and health are lacking, specifically when animals are experiencing severe disease challenges. Furthermore, the impact of the inability to treat sick animals with ABs on the feelings and emotions of farm personnel has not been evaluated. It is well documented through work in the social sciences that there are barriers and conflicts to the use of antimicrobials that require resolution before certain practices can be adopted. For example, social approval as it pertains to antibiotic use and animal welfare is important to farmers, who are innately insecure as to how they are perceived by society [20]. In addition, animal health status and management quality have been determined to be important factors that influence farmers’ decision-making concerning antibiotic use [21].

Therefore, the purpose of this study was to compare production performance and health parameters in pigs raised in conventional systems which practice responsible use of ABs or ABF systems, in the face of an acute disease challenge with porcine reproductive and respiratory syndrome virus (PRRSV). Due its significant economic impact across the US swine industry [22], PRRSV was selected as the challenge agent. In addition, due to its industry prevalence and high degree of pathogenicity, PRRSV 174 was selected as the challenge variant [23]. The study was based on the hypothesis that the responsible use of ABs will effectively improve the performance and health of sick animals.

## Materials and methods

### Welfare statement

Throughout the study, animal health and welfare standards were maintained in accordance with the institutional animal care and use guidelines observed by the investigators’ ethical review boards (Zoetis IACUC trial number 16TDSBIOPORK01 and Pipestone Applied Research IACUC trial number 17–4). Both IACUC organizations reviewed and approved the trial protocol and mortality standards prior to its initiation. Pigs were observed on a daily basis by animal husbandry personnel under the supervision of an attending veterinarian. Throughout the entire trial period, sick pigs were given appropriate treatment and transferred to a designated hospital pen when appropriate. Pigs with no prospects for recovery (unable to ambulate, eat, drink, etc.) and those with no evidence of health status improvement after 3 days of treatment were removed from the trial and humanely euthanized. During the pre-weaning phase of the trial, pigs were euthanized by blunt force trauma if < 5 days of age, or by electrocution if > 5 days of age. In the post-weaning phase, pigs with no prospects for recovery (unable to ambulate, eat, drink, etc.) with no evidence of health status improvement after 3 days of treatment were removed from the trial and humanely euthanized via penetrating captive bolt. Euthanasia was performed only by qualified personnel that had undergone training supervised by the Pipestone welfare department.

### Source of animals

Test pigs were sourced from a 5000 sow breeding unit in eastern South Dakota, USA, consisting of commercial, cross-bred (Large White x Landrace) sows that were, diagnostically confirmed to be PRRSV-naive and *Mycoplasma hyopneumoniae*-stable. This unit was mechanically ventilated and filtered incoming air to reduce the risk of airborne pathogen introduction. Personnel entered the unit via a shower-in protocol. Sows were bred using artificial insemination and housed in stalls during pregnancy and in maternity pens while nursing. Environmental comfort (heat mats and lamps) were provided for piglets, based on observed behavior (piling if chilled, etc.). The unit was visited monthly by Pipestone veterinarians, production supervisors, welfare auditors and biosecurity technicians. Unannounced third-party welfare audits were conducted quarterly.

### Experimental design: Pre-weaning protocols

**Fig 1:**
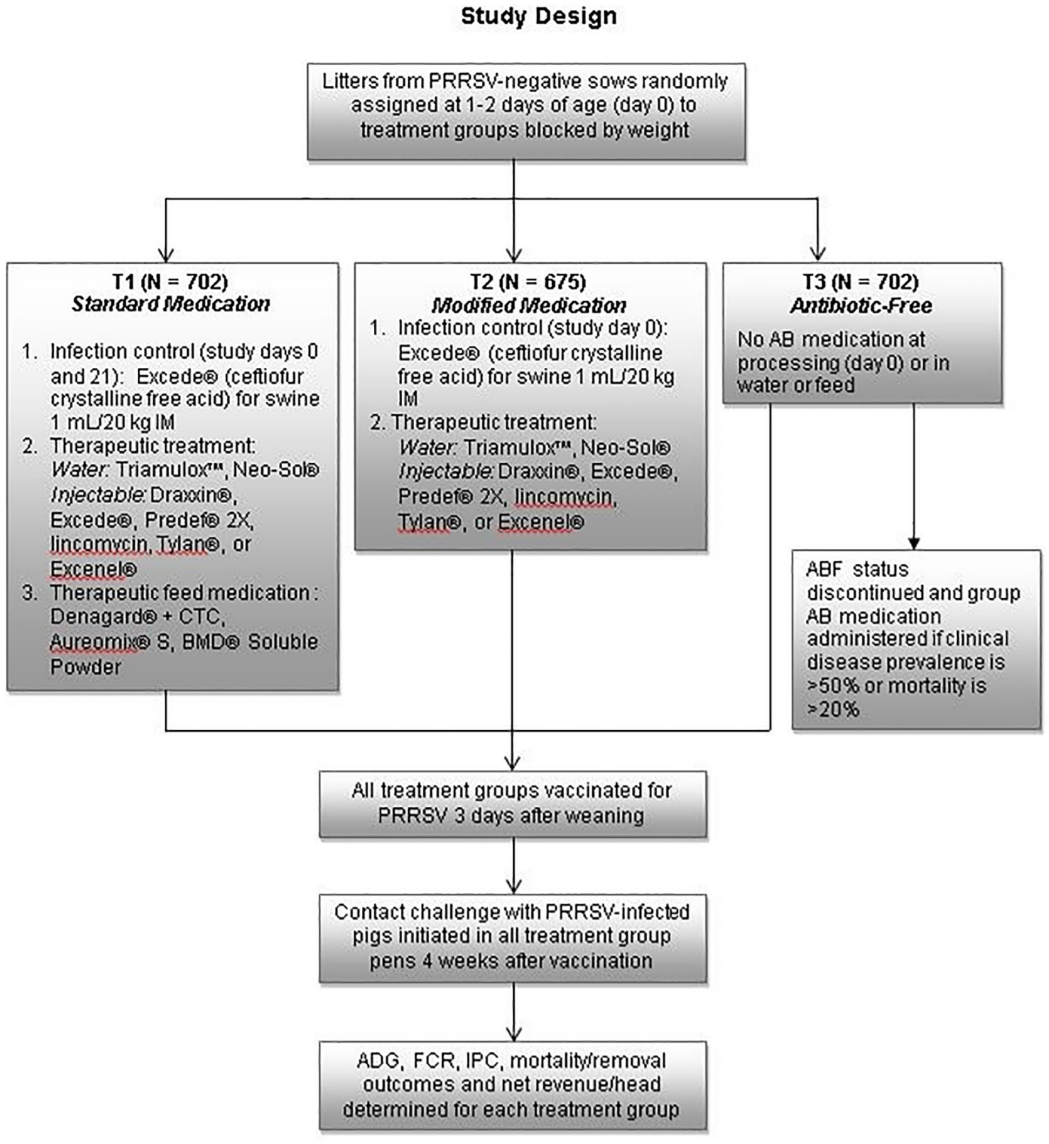
Study design. Outcomes were compared for pigs managed with standard AB medication (T1), modified AB medication (T2), or an antibiotic-free (ABF) regimen (T3). AB = antibiotic; ADG = average daily gain; FCR = feed conversion ratio; IPC = Individual Pig Care; PRRSV = porcine reproductive and respiratory syndrome virus.

The trial was a randomized, controlled study. A total of 208 sows and litters were randomly selected via computer to obtain the required number of animals for the study (~2100). Test groups were allocated to one of 3 treatment groups (Fig 1). Treatment was applied to the litter in an alternating pattern taking into account sow parity. Specifically, the first sow enrolled was treatment A, the second sow enrolled was treatment B, the third sow enrolled was treatment C, and the process was repeated until enrollment was completed. Parity 1 sows were enrolled separately from parity 2+ sows. On trial day 0 (day 1–2 of age, following completion of cross-fostering), litters were sorted into small (< 1.4 kg), medium (1.4–1.7 kg), and large (> 1.7 kg) weight categories. An attending veterinarian confirmed that all enrolled pigs were clinically healthy. Each pig was uniquely identified at enrollment with duplicate ear tags color-coded by treatment group and attached in opposite ears, individually weighed, and identified by sow, parity, and gender. Pigs in treatment group T1 (N = 702) received a standard medication protocol of ABs given on days 4 of life (processing) and 21 (weaning) consisting of Excede (ceftiofur crystalline free acid, Zoetis, Parsippany, NJ) via the IM route and subsequent therapeutic ABs as needed administered via the IM route, or through the water and feed. Pigs in treatment group T2 (N = 702) received a modified medication protocol consisting of Excede on day 4 and subsequent therapeutic ABs as needed, via the IM route and the water (no feed). All medications were given per label instructions. Pigs in treatment group T3 (N = 702) were considered antibiotic free and received no infection control or therapeutic ABs via the IM route, water or feed, although anti-inflammatory, analgesic or antipyretic products, including dexamethasone and flunixin meglumine (Banamine, Merck, Madison, NJ). All test pigs were vaccinated with a porcine circovirus type 2 (PCV2)-*M*. *hyopneumoniae* combination vaccine (Fostera PCV MH, Zoetis) one day prior to weaning and 3 weeks thereafter. At weaning, pigs weighing < 3.6 kg or that were lame, deformed, or clinically sick were removed from the study and humanely euthanized via blunt force trauma. Radio Frequency Identification (RFID) tags were affixed to the ears of all remaining pigs.

### Experimental design: Post-weaning protocols

The experimental unit was the pen. The trial utilized a randomized block design, sorting piglets by size. A total of 26 blocks were used in the study. Each block consisted of 1 of the 3 sizes of pigs, (Small, Medium, and Large) and involved 3 consecutive neighboring pens in the barn. At 21 days of age pigs were moved to a Pipestone Applied Research wean-to-finish facility in southwest Minnesota for the remainder of the trial. This facility included 78 test pens providing 26 pens per treatment, based on a sample size calculation (alpha = 0.05, power = 0.8 and standard deviation = 0.12) which allowed for detection of a difference of 0.04 kg between treatments T1, T2 and T3. Twenty-seven pigs were placed in each pen, allowing for .09 square meter of space per pig from weaning to shipment to the harvest facility. Pigs were housed in pens according to treatment group and size and each pen represented 7–10 litters. Each pen had a 4-hole dry feeder which provided 35 cm of feeder space per pig (6.75 pigs per feeder hole) and 2 cup waterers per pen. Feed and water was provided *ad libitum*. Pigs were fed using an automated delivery system for measuring feed consumption (Feedlogic Corporation, Willmar, MN). Water consumption was monitored on a daily basis using water meters. Feed consumption was recorded weekly for each treatment group. Samples obtained from each feed delivery from the feed truck compartments and the Feedlogic delivery system were analyzed to confirm ABF-status or AB inclusion rates (analyses performed by the Customer Analytics Support Department, Zoetis, Chicago Heights, IL). Barrows and gilts were penned separately and no environmental enrichments were provided to any pens in the facility. Approved water medications for T1 and T2 pigs included neomycin sulfate (Neo-Sol 50, Alpharma, Huvepharma, Sophia, Bulgaria), tiamulin hydrogen fumarate (Triamulox, Zoetis), and sulfadiazine-trimethoprim (Equisol-SDT, Aurora, Northfield, MN). No feed medication was provided to the T2 group. Pigs were also vaccinated for PRRSV (Fostera PRRS, Zoetis) 3 days after weaning. Pigs in treatment group T3 (ABF) received no AB treatment; however, anti-inflammatory, analgesic or antipyretic supportive therapy was provided. This included IM dexamethasone and flunixin meglumine (Banamine, Merck, Madison, NJ) and oral buffered electrolytes (Sky-Lytes, Skylabs, Rushville, IL). All medications were given per label instructions.

### Challenge agent and procedure

To mimic natural infection dynamics, a seeder pig model was used to challenge pigs in each treatment group with PRRSV variant 174. Challenge occurred 4 weeks after PRRSV vaccination. To initiate challenge, an IM dose of 2 x 10^3.5^ TCID_50_ PRRSV lineage 1 strain 174 was administered to 3 randomly selected seeder pigs in each pen, representing approximately 10% of the pen population. Seeder pigs remained in continuous contact post-challenge with their pen mates until the conclusion of the trial or until they were removed due to death or treatment failure. Due to the level of pathogenicity of this variant, at any time during the trial should the attending veterinarian determine that > 50% of pigs in the ABF group showed evidence of infectious disease that failed to respond to therapy or if group mortality was > 20%, the ABF component of the study would cease, mass-medication would be applied, and the study would continue.

### Outcomes measured

#### Performance outcomes

Average daily gain (ADG) and feed conversion ratio (FCR) were calculated for each treatment group on days 0, 28, 49, 70, and 147. Day 147 outcomes included the percentage of full-value pigs (defined as pigs weighing > 105 kg at marketing), the percentage of those with defects including intact males, lame animals, animals with umbilical hernias, the percentage of lightweight pigs (< 105 kg at marketing), and the percentage of mortalities and removals. In both the pre-weaning and post-weaning periods, removals were defined as animals from the T3 ABF group that had been treated with antibiotics, as well as any animal from groups T1 and T2 that had been removed to a hospital pen as previously defined.

#### Clinical scoring

Individual Pig Care (IPC) scoring for determination of animal health status (IPC, Zoetis) was performed for all pigs every 3 days for the first 3 weeks after weaning, and after challenge 3 times weekly for 3 weeks and then once weekly until the end of the trial. Scoring was performed by personnel trained in the IPC method [24,25]. IPC scores were calculated using an A-B-C scoring system with “A” referring to mild acute disease, “B” referring to moderate clinical disease, and “C” referring to severe clinical disease.

#### Personnel monitoring

To monitor the human component throughout the study, husbandry caregivers were surveyed each week of the study for a response to the question ‘Are you satisfied with the efficacy of the care being given to the study animals?’ Their level of agreement was scored on a 10-point scale from 1 (full agreement) to 10 (no agreement) for each of the treatment groups.

#### Economic analysis

In order to estimate the potential economic effect of PRRSV infection across the 3 groups, a Net Revenue calculation was performed using the following equation:

Netrevenue=(Finalweight/100x$56/cwt)–mortalityloss+valueofdefectcullsandlightweightpigs.

For the purpose of the analysis, mortality loss was set at $35 per death plus an estimate of the number days on feed prior to dying, using an estimated feed intake of $0.24/kg feed, not inclusive of feed medication costs. In addition, the value of defect culls and lightweight pigs were set at 60% and 90% of full value pigs, respectively. The cost of vaccines and injectable medications were not included in the analysis.

### Diagnostic procedures

Diagnostic procedures for PRRSV infection consisted of necropsies performed on a subset of mortalities occurring 1 to 4 weeks after challenge. PRRSV infection was confirmed by polymerase chain reaction (PCR) testing along with nucleic acid sequencing of the open reading frame 5 segment to confirm the presence of PRRSV 174. In addition, oral fluid samples were collected every 4 weeks after challenge to determine the presence of PRRSV and other pathogens, including rotavirus A, B, and C, influenza A virus of swine (IAV-S), PCV2, and *M*. *hyopneumoniae* by the Animal Disease Research and Diagnostic Laboratory, South Dakota State University. Randomly selected mortalities were submitted to Iowa State University Veterinary Diagnostic Laboratory for culture and sensitivity to evaluate the presence of secondary bacterial pathogens.

### Statistical analysis

Statisticians were blinded to the identity of the treatment groups to which test animals were assigned. For the wean-to-finish analysis, weight, ADG, average daily feed intake, and FCR were analyzed by a linear mixed model approach for repeated measures (RLMM). Using the SAS Proc Mixed Procedure (SAS 9.4, Cary NC), all of these variables were analyzed with a model that considered the fixed effects of treatment, day and the interaction of treatment-by-day and the random effects of room, block (room) and the residual error. The pen was the experimental unit and treatment day was the repeated factor. The data were checked for normality before the analysis. The covariance structure in the repeated measures analysis was investigated using 6 structural assumptions, namely compound symmetry, heterogeneous compound symmetry, power, first-order autoregressive, heterogeneous first order autoregressive, and unstructured. The assumption giving the minimum value of the Akaike’s Information Criterion was selected in the final analysis. Treatment least square means (LSMeans) were calculated for each group. Comparisons of LSMeans were performed by the two-sided Student’s t-test at the 5% level of significance. Treatment and treatment-by-day effects were assessed at the 5% level. If the treatment-by-day interaction was significant, then treatment comparisons were assessed for each treatment group within each day at the 5% level of significance. Full value, defects, lights and mortality/removals were defined as binary outcomes (1 = yes, 0 = no) and analyzed using a generalized linear mixed model approach. Using the SAS Glimmix procedure, these variables were analyzed with a model that considered the initial weight as a covariate, the fixed effect of treatment and the random effects of room, block (room) and the residual error. This analysis utilized a binomial error and logit link. Glimmix results were back-transformed. A p-value of ≤0.05 was used in all tests as a criterion for statistical significance.

For the farrow-to-wean analysis, enrollment weight, farrow-to-wean ADG, weaning weight, and wean standard deviation, the analysis utilized a model with the fixed effects of treatment, litter size and parity, and the random effect of room. LSMean comparisons were performed using the same methodology as the wean-to-finish analysis. Farrow-to-wean mortality/removals were defined as binary outcomes and analyzed with a model that considered the fixed effects of treatment, litter size and parity, and the random effect of room. This analysis utilized a binomial error and logit link. Glimmix results were back-transformed. A p-value of ≤ 0.05 was used in all tests as a criterion for statistical significance.

## Results

### Clinical signs and diagnostic data

Three days post-challenge, an attending veterinarian observed clinical signs, including pyrexia and dyspnea in all seeder pigs. Seven days post-challenge, blood samples from 10 pigs from each treatment group confirmed PRRSV-positive by PCR with Ct values ranging from 16.4–18.6. Open reading frame 5 sequencing confirmed that the positive samples were closely related (99.5–100% homology) to the PRRSV 174 used in the inoculum. Eight days after challenge, a total of 9 clinically sick pigs were randomly selected from each of the 3 study groups (3 pigs per group), blood tested, euthanized, and necropsied. PRRSV 174 was isolated from the lungs of 9 pigs, while 8 of the pigs had interstitial pneumonia with necrotic alveolar macrophages, typical of PRRSV infection. All blood samples were serologically positive by ELISA (IDEXX, PRRS X3, Westbrook, ME). In addition, the following pathogens were identified by direct culture or PCR from several tissues across these pigs including, *Haemophilus parasuis*, *Streptococcus suis*, *Pasteurella multocida*, *Salmonella cholerasuis*, *Bordetella bronchiseptica*, *Mycoplasma hyosynoviae*, *Mycoplasma hyorhinis*, *E*. *coli*, influenza virus A of swine, porcine circovirus type 2 and rotavirus A, B and C.

### Performance outcomes

#### Pre-weaning

Pigs in the 3 treatment groups had comparable mean enrollment weights (Table 1, p≥0.05), indicating that randomization resulted in test groups without significant compositional differences. Pre-weaning performance (Table 1) indicated that T3 pigs had significantly lower mean weaning weights and ADG and a significantly greater mean mortality and removals as compared to the T1 and T2 groups. Most notably, T3 mortality and removal rate was 2.4-fold greater than that for T1 pigs and 2.9-fold greater than that for T2 pigs. In regards to the actual number of mortalities and removals during the lactation period, there were 46 mortalities and 1 removal in group T1, 39 mortalities in group T2 (0 removals), and 56 mortalities and 71 removals in group T3. In total, across the pre-weaning phase of the study, a total of 141 pigs died, including 46 pigs in T1, 39 pigs in T2 and 56 pigs in T3.

**Table 1:** Pre-weaning performance comparison in litters raised using a standard (T1), modified antibiotic medication (T2), or ABF production protocol (T3).

Production Parameter	T1 (LSMean ± SEM)	T2 (LSMean ± SEM)	T3 (LSMean ± SEM)
# Litters	57	56	56
Enrollment Weight (kg)	1.57^a^ ± 0.051	1.58^a^ ± 0.055	1.51^a^ ± 0.053
Weaning Weight (kg)	5.97^a^ ± 0.158	5.90^a^^b^ ± 0.162	5.62^b^ ± 0.163
Averaged daily gain at weaning (kg)	0.21^a^ ± 0.006	0.21^a^ ± 0.006	0.20^b^ ± 0.006
Mortality and Removals (%)	7.21^b^ ± 3.051	6.48^b^ ± 2.788	16.55^a^ ± 5.959

^a, b^Values with different superscripts in the same row are statistically different (p ≤ 0.05).

#### Post-weaning

In regard to post-weaning performance, compared to the T3 group, ADG was significantly greater for the T1 and T2 groups at finishing and at each post-weaning time point except for the interval from day 71–147 (Table 2). There were no significant differences in post-weaning ADG between the T1 and T2 groups. Overall, the FCR results favored T1 and T2, both which were significantly different than that of T3. Most notably, the FCR for the day 29–49 interval reflected the negative ADG for T3 pigs at the same production phase (Table 2).

**Table 2:** Average daily gain and feed conversion ratio (FCR) by production phase and treatment group. Data are presented as the LSmean ± SEM. Removal weights were not added back into the pen weight for the ADG and FCR calculations.

	Day 0–28	Day 29–49	Day 50–70	Day 71–147	Day 0–147
ADG					
T1	0.35^a^ ± 0.016	0.21^a^ ± 0.023	0.66^a^ ± 0.022	0.89^a^ ± 0.013	0.63^a^ ± 0.012
T2	0.34^a^ ± 0.016	0.15^a^ ± 0.023	0.67^a^ ± 0.023	0.90^a^ ± 0.013	0.62^a^ ± 0.013
T3	0.27^b^ ± 0.016	-0.15^b^ ± 0.023	0.52^b^ ± 0.023	0.91^a^ ± 0.013	0.46^b^ ± 0.013
FCR					
T1	0.61^b^ ± 0.065	0.71^a^ ± 1.728	0.83^a^ ± 0.443	1.22^a^ ± 0.012	1.10^b^ ± 0.015
T2	0.66^b^ ± 0.065	0.43^a^ ± 1.769	0.80^a^ ± 0.454	1.19^a^ ± 0.012	1.10^b^ ± 0.015
T3	0.85^a^ ± 0.065	-4.90^b^ ± 1.773	0.37^a^ ± 0.455	1.19^a^ ± 0.012	1.27^a^ ± 0.015

^a,b^Values with different superscripts in the same row are statistically different

### Clinical outcomes

**Fig 2:**
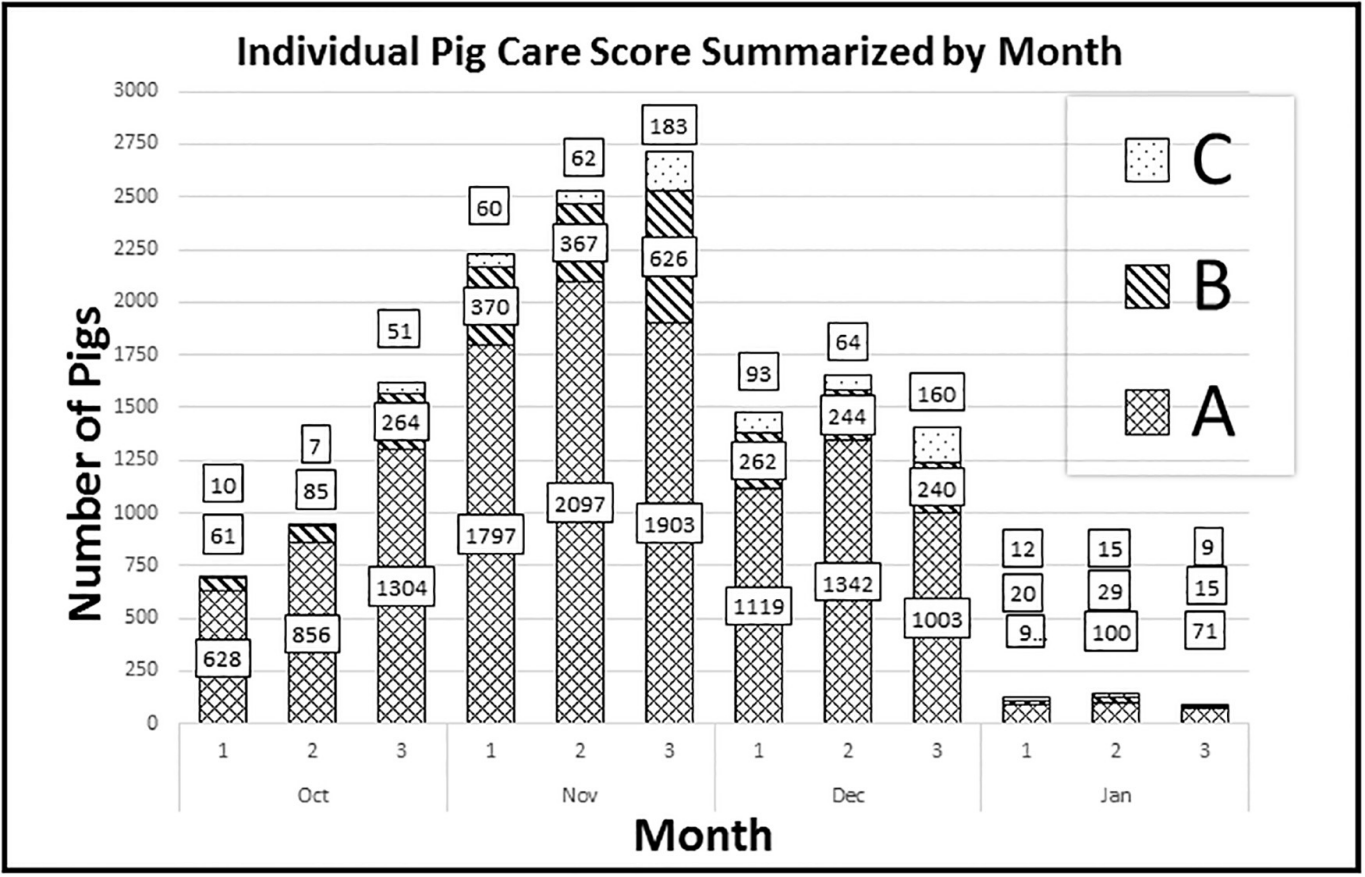
Individual Pig Care (IPC) scores for treatment groups T1, T2, and T3 during the four months from weaning to finish. The three numerical values adjacent to each bar indicate, in descending order, the IPC scores for categories A (mild clinical disease), B (moderate clinical disease), and C (severe clinical disease). IPC scores spiked immediately after PRRSV challenge (C arrow) and declined when ABF restrictions were lifted and group medication was initiated (M arrow) for all groups, including the antibiotic-free group T3 as an animal-health intervention. The standard medication group T1 had the lowest cumulative IPC scores before PRRSV challenge and during the month following challenge, indicating that this group had the lowest level of clinical disease, followed successively by the T2 and T3 groups.

The IPC scores (Fig 2) provided descriptive data summarizing the incidence and severity of clinical disease across treatment groups. Category A scores predominated at all wean-to-finish time points for each group, indicating that non-specific clinical disease was a largely treatable, high-morbidity, low-mortality condition. Category B and C cases of increased severity were relatively less common than A scores at each time point. IPC scores spiked in all treatment groups during November, following challenge. The ABF group (T3) had the highest combined IPC scores, not only following PRRSV challenge, but in the month preceding challenge, consistent with the relatively high mortality rate before and after weaning. In total 631 pigs died during the post-weaning phase of the trial across all 3 groups with 426 found dead and 205 humanely euthanized.

Clinical signs of infectious respiratory or enteric disease were present in >50% of the T3 pigs within 4 weeks after challenge and mortality in this group exceeded 20%. As a result, ABF status was discontinued. According to culture and sensitivity results, ceftiofur and sulfadiazine-trimethoprim were administered to the entire population, via the IM route and the water, respectively. Response to mass medication produced a favorable response and over time, IPC scores diminished to negligible levels for all treatment groups. Final marketing results across all 3 groups are provided in Table 3. Across the 3 groups, the percentage of full value pigs marketed was significantly greater in groups T1 and T2 (68.09% and 65.33%) when compared to group T3 (33.05%). In addition, the percentage of mortalities and removals was significantly greater in group T3 (57.98%) when compared with groups T1 and T2 (20.94% and 24.89%). The actual number of pigs removed from the study and placed in a designated hospital pen, and the actual number of pigs that either died or were euthanized across all 3 groups was as follows: T1: 15 removals and 132 mortalities, T2: 23 removals and 152 mortalities and T3: 60 removals and 347 mortalities. In contrast, there were no significant differences among the 3 test groups in percentages of defective pigs or light-weight pigs and percentages of full-value pigs and mortality and removals were not significantly different between the T1 and T2 groups (Table 3).

**Table 3:** Final market results for litters raised using a standard (T1) or modified (T2) antibiotic medication or an antibiotic-free (T3) production protocol.

Production Parameter	T1 (LSMean ± SEM)	T2 (LSMean ± SEM)	T3 (LSMean ± SEM)
% Full value pigs	67.36^a^ ± 1.809	64.27^a^ ± 1.895	34.99^b^ ± 1.896
% Defects	1.12^a^ ± 0.419	1.03^a^ ± 0.407	0.74^a^ ± 0.351
% Light-weight pigs	9.83^a^ ± 1.179	8.90^a^ ± 1.155	6.97^a^ ± 0.989
Mortality and removals (%)	21.26^b^ ± 1.584	25.36^b^ ± 1.729	56.73^a^ ± 2.001
Net revenue ($)*	74,014	66,682	23,735
Net revenue/pig ($)*	105.43	98.79	33.81

^a, b^Values with different superscripts in the same row are statistically different (p ≤ 0.05).

*Net revenue = Final weight/100 x $56/cwt–mortality loss. Mortality loss = $35/death + no. days on feed x estimated feed intake x $0.24/kg feed. Defect culls = 60% of full-value pigs; light-weight pigs = 90% of full-value pigs.

### Economic analysis

Using the net revenue calculation previously described, net revenue per pig was 3.1-fold greater for the T1 vs. T3 pigs ($105.43 vs. $33.81) and 2.9-fold greater for the T2 vs. T3 pigs ($98.79 vs. $33.81). The net revenue per pig was $6.64 greater for the T1 group compared to T2 pigs. These data are summarized in Table 3.

### Personnel monitoring

**Fig 3:**
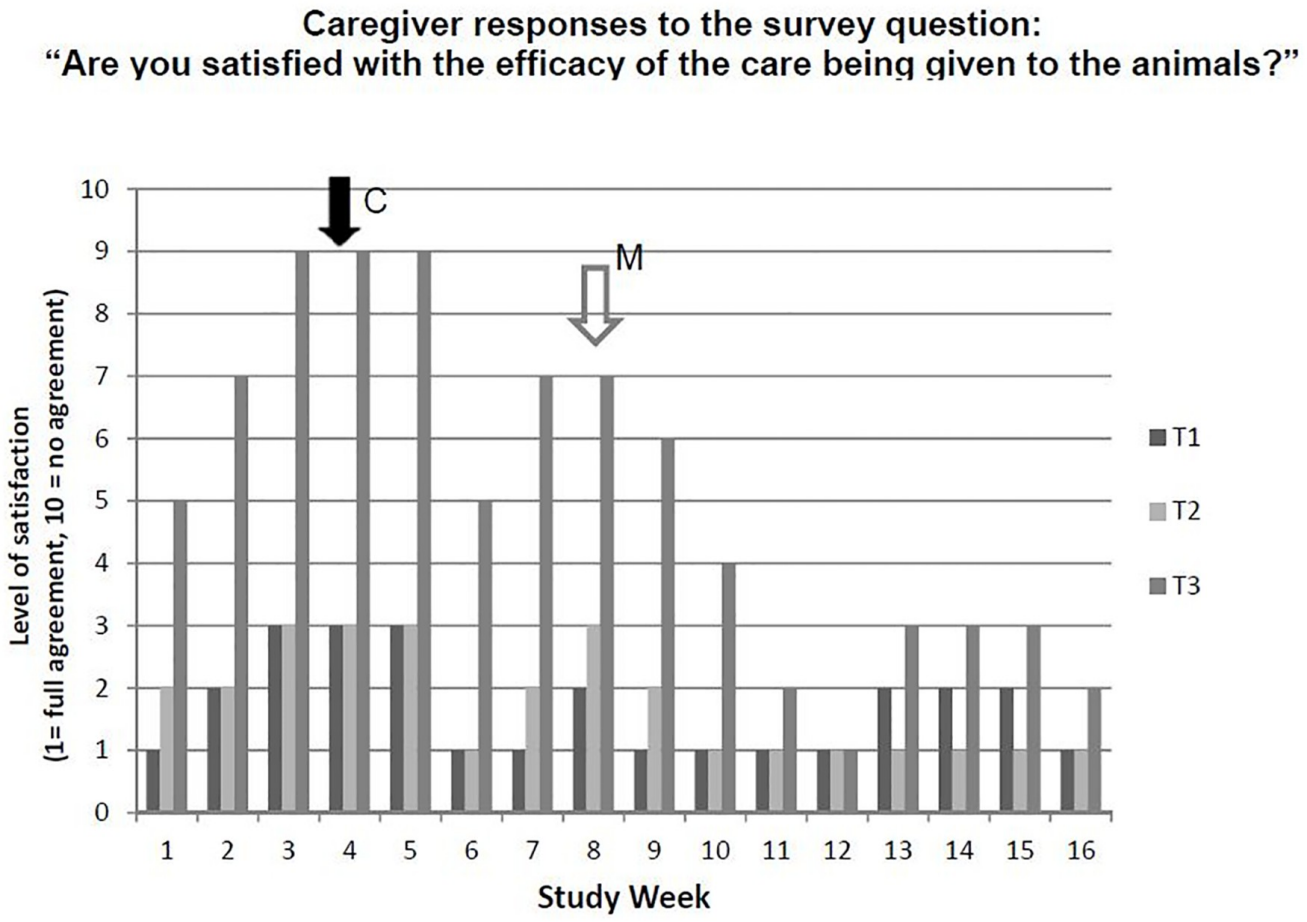
Caregiver responses to the survey question ‘Are you satisfied with the efficacy of the care being given to the study animals?’. Response are shown as mean values on a 10-point scale from 1 (full agreement) to 10 (no agreement). Responses were based on the observed health status of pigs in each treatment group at each week of the study. For the first 11 weeks of the study, satisfaction scores were most favorable for T1 and T2 pigs at each time point compared to scores for ABF (T3) pigs. Relatively favorable scores for the T1 and T2 groups reflected the absence of clinical disease due to infection control treatment on day 0 or subsequent therapeutic AB treatment, which was denied in T3 pigs. C = challenge with PRRSV; M = group medication of pigs in all three groups, including T3 pigs.

Results of caregiver responses to the survey question ‘Are you satisfied with the efficacy of the care being given to the study animals?’ are shown in Fig 3. For the first 11 weeks of the study, health satisfaction scores were most favorable for T1 and T2 pigs at each time point compared to scores for ABF (T3) pigs. Relatively favorable scores for the T1 and T2 groups reflected the absence of clinical disease in pigs given injectable infection control medication on day 0 or therapeutic AB treatment, which was denied in T3 pigs. The least favorable health satisfaction scores occurred just before and after PRRSV challenge. Scores began to improve in the T3 group following group AB treatment initiated at week 8 of the trial.

### Assays for antibacterial agents in feed

The feed concentrations of AB agents fed to group T1 were within label specifications for chlortetracycline (CTC, 400 g/ton), Denagard (35 g/ton), Aureomycin (400 g/ton) and BMD (30 g/ton). The concentration of CTC and sulfamethazine (<2.0 g/ton) in Aureomix S fed to group T1 was less than label specifications (100 g/ton for each ingredient). All samples fed to groups T2 and T3 were free of detectable levels of AB agents except for two deliveries, which had trace levels of CTC (3.2 and 11.5 g/ton, respectively).

## Discussion

As stated previously, there has been an increased public concern regarding the role of AB use in agricultural and what role that could/may play in the development of antimicrobial resistance in humans [1–4]. This has resulted in increased interest in the ability to raise livestock under antibiotic-free conditions to decrease AB use in farm animals. However, prior to this study, no data were available regarding the production performance and health of animals raised ABF when under a significant disease challenge such as PRRSV, the most significant pathogen in the global swine industry. While PRRS is a viral disease, due to its immunosuppressive capabilities, the effect of secondary bacterial pathogens on infected pigs is significant and requires the ability of the veterinarian to use ABs in a responsible manner to treat affected animals. Therefore, the objective of this trial was to generate information on the production performance and animal health parameters in pigs raised in conventional systems which allowed the use of ABs to treat sick animals or ABF.

Under the conditions of the study, pig performance and health were significantly different when responsible use of ABs were permitted as compared to those seen in the ABF system, throughout the pre-weaning and post-weaning phases of the trial. The benefits of responsible AB use was further reflected by a final removal/mortality rate in ABF pigs that was more than double the rates in T1 and T2 pigs, and net revenue per pig that was less than a third of that seen when pigs were treated with ABs. In addition, following termination of the ABF (T3) group, performance of the entire population improved and differences in ADG were not observed during the latter stages (day 71–147) of the trial.

The caregiver observational scoring (Fig 3) was consistent with the IPC scores and patterns of clinical disease: T1 and T2 pigs fared better than their ABF counterparts in the estimation of husbandry personnel; however, the caregiver scoring was subjective and not evaluated statistically. Despite this limitation, it captured a valid aspect of food animal production, namely that humane production methods are consistent with avoidance of disease and optimum productivity outcomes [26]. As such, interventions such as judicious AB use are important components of livestock production in North America and elsewhere [27]. It is for this reason that systematic, animal health-based approaches to pork production, such as the National Pork Board’s Pork Quality Assurance and Swine Welfare Assurance programs, are widely practiced in the U.S. and elsewhere.

As with all experiments, this trial had both strength and limitations. Strengths included the randomized, controlled format, blinded statistical analysis of results, and evaluation of multiple metrics (production values, measures of mortality and clinical disease). The large study population supported the statistical power of the results. The test population was challenge-exposed to a representative strain of viral pathogen that is endemic in most swine-producing regions and has proven to have a substantial adverse economic impact, approximating actual field conditions previously described [23]. Limitations of the trial were evaluation of only a single production turn, along with the use of a highly pathogenic variant of PRRSV, as an isolate of less virulence may have generated different results, and the absence of negative controls in the form of non-challenged medicated and ABF groups.

In conclusion, while interest in ABF production strategies may be increasing in the global swine industry and may be feasible when applied to high-health herds in areas of low disease prevalence, results of this trial clearly demonstrate the significant limitations of ABF production under high conditions of disease challenge. In addition, results indicate that the judicious use of ABs during active disease outbreaks is essential to protect the health and well-being of animals. Further efforts to protect the ability to use ABs responsibly in livestock populations should be a major focus of veterinarians and industry professionals. Losing these important tools through efforts to reduce use would significantly reduce the ability to provide wholesome, safe products to feed the global population. It is the authors’ hope that the lessons learned from this project will help facilitate the continued ability to practice responsible AB use. This practice, in conjunction with the adaptation of efficacious disease prevention strategies [28] to protect health status and minimize the need to use ABs, will help to maximize their efficacy and reduce the risk of antimicrobial resistance over time.

## Supporting information

S1 File Sow data ABF kg. (XLSX)

S2 File Weights ABF kg. (XLSX)

S3 File Feed ABF kg. (XLSX)

S4 File Mortality ABF. (XLSX)

S5 File Arrive guidelines checklist D18-10826. (PDF)
